# Structural basis of nucleosomal H4K20 recognition and methylation by SUV420H1 methyltransferase

**DOI:** 10.1038/s41421-023-00620-5

**Published:** 2023-12-05

**Authors:** Folan Lin, Ruxin Zhang, Weihan Shao, Cong Lei, Mingxi Ma, Ying Zhang, Zengqi Wen, Wanqiu Li

**Affiliations:** 1 Department of Pharmacology, Joint Laboratory of Guangdong-Hong Kong Universities for Vascular Homeostasis and Diseases, School of Medicine, Southern University of Science and Technology, Shenzhen, Guangdong China; 2 Institute of Molecular Physiology, Shenzhen Bay Laboratory, Shenzhen, Guangdong China; 3 School of Medicine, Shenzhen Campus of Sun Yat-Sen University, Sun Yat-Sen University, Shenzhen, Guangdong China; 4 Institute for Biological Electron Microscopy, Southern University of Science and Technology, Shenzhen, Guangdong China

**Keywords:** Histone post-translational modifications, Cryoelectron microscopy, Nucleosomes

## Abstract

Histone lysine methyltransferase SUV420H1, which is responsible for site-specific di-/tri-methylation of histone H4 lysine 20 (H4K20), has crucial roles in DNA-templated processes, including DNA replication, DNA damage repair, and chromatin compaction. Its mutations frequently occur in human cancers. Nucleosomes containing the histone variant H2A.Z enhance the catalytic activities of SUV420H1 on H4K20 di-methylation deposition, regulating early replication origins. However, the molecular mechanism by which SUV420H1 specifically recognizes and deposits H4K20 methyl marks on nucleosomes remains poorly understood. Here we report the cryo-electron microscopy structures of SUV420H1 associated with H2A-containing nucleosome core particles (NCPs), and H2A.Z-containing NCPs. We find that SUV420H1 makes extensive site-specific contacts with histone and DNA regions. SUV420H1 C-terminal domain recognizes the H2A–H2B acidic patch of NCPs through its two arginine anchors, thus enabling H4K20 insertion for catalysis specifically. We also identify important residues increasing the catalytic activities of SUV420H1 bound to H2A.Z NCPs. In vitro and in vivo functional analyses reveal that multiple disease-associated mutations at the interfaces are essential for its catalytic activity and chromatin state regulation. Together, our study provides molecular insights into the nucleosome-based recognition and methylation mechanisms of SUV420H1, and a structural basis for understanding SUV420H1-related human disease.

## Introduction

As the building block of chromatin, the nucleosome is composed of 147 bp DNA wrapped around a compact histone octamer^1^, where the core histones possess a multiplicity of post-translational modifications (PTMs)^2^. Histone lysine methylation is a unique PTM because of its relative stability and multivalency, and plays a crucial role in a wide variety of DNA-templated processes including transcription, replication, and DNA repair^3,4^. The canonical lysine methylation mark on histone H4 at lysine 20 (H4K20) is conserved from yeast to humans. Histone H4 lysine 20 methylation (H4K20me) is a crucial modification for genome maintenance involved in replication, DNA damage repair, and chromatin compaction^5–10^. There are three distinct H4K20 methylation states: mono- (H4K20me1), di- (H4K20me2), and tri-methylation (H4K20me3), associated with different biological functions. It is known that SET8 (also known as PR-Set7) is responsible for the H4K20 mono-methylation (H4K20me1), whereas SUV420H1 and SUV420H2 catalyze H4K20 di-methylation (H4K20me2) and tri-methylation (H4K20me3), respectively. H4K20me1 and H4K20me2 are important in DNA replication and DNA repair, whereas H4K20me3 is a hallmark of silent heterochromatin. Studies have shown that H4K20me2 as the most abundant methylation state on histone H4 is widely distributed across the genome^9,11,12^, indicating its crucial role in DNA-templated processes. Dysregulation of H4K20 methylation states leads to various diseases, including cancer^13^.

SUV420H1 is a member of the SUV4-20H family protein, responsible for catalyzing the majority of H4K20me2 and H4K20me3 modifications. This family consists of a unique N-terminal domain, a catalytic SET domain, and a Zn-binding post-SET domain. SUV420H1 mediated H4K20me2 has been linked to the recruitment of 53BP1 to DNA double-strand breaks regulating DNA damage repair^7,10,14^. H4K20me2 is also enriched at replication origins, and abrogating ORC1 recognition of H4K20me2 in cells impairs ORC1 occupancy at replication origins^6^. A recent study identified that histone variant H2A.Z promotes the catalytic activities of SUV420H1 on H4K20me2 mark deposition, which in turn facilitates ORC1 recruitment, regulating DNA replication^15^. Based on the catalog of somatic mutations in cancer (COSMIC) database, somatic mutations of SUV420H1 frequently occur in different types of cancers^16^. In addition, it has been shown that SUV420H1 dysregulation is associated with neurodevelopmental abnormalities^17–19^.

Several somatic cancer mutations in SUV420H1 exhibited lower enzymatic activity on nucleosomes^20^. While the structure of SUV420H1 is currently limited to the SET domain^21^. Recently, significant advancements in the structures of major histone methyltransferases in complexes with nucleosomes have provided vast insights into their enzymatic mechanisms and the relevance of diseases^22–25^. However, the mechanism by which SUV420H1 specifically recognizes and methylates H4K20 at the nucleosome level remains largely unclear. Additionally, the mechanism underlying the enhancement of SUV420H1 activities by H2A.Z-containing nucleosomes remains puzzling.

Here, we report the cryo-electron microscopy (cryo-EM) structures of SUV420H1 bound to H2A-containing nucleosome (SUV420H1–NCP^H2A^) and H2A.Z-containing nucleosome (SUV420H1–NCP^H2A.Z^) at an overall resolution of 3.58 Å and 3.68 Å, respectively. In combination with structure-guided functional experiments, our results provide an overview of how SUV420H1 interacts with the histones and DNA components of the nucleosome to specifically recognize and catalyze H4K20. Our results also elucidate how H2A.Z promotes SUV420H1 catalytic activity.

## Results

### Biochemical analysis of SUV420H1 and the overall structure of the SUV420H1_(1__–__390)_–NCP^H2A.Z^ complex

**Fig. 1:**
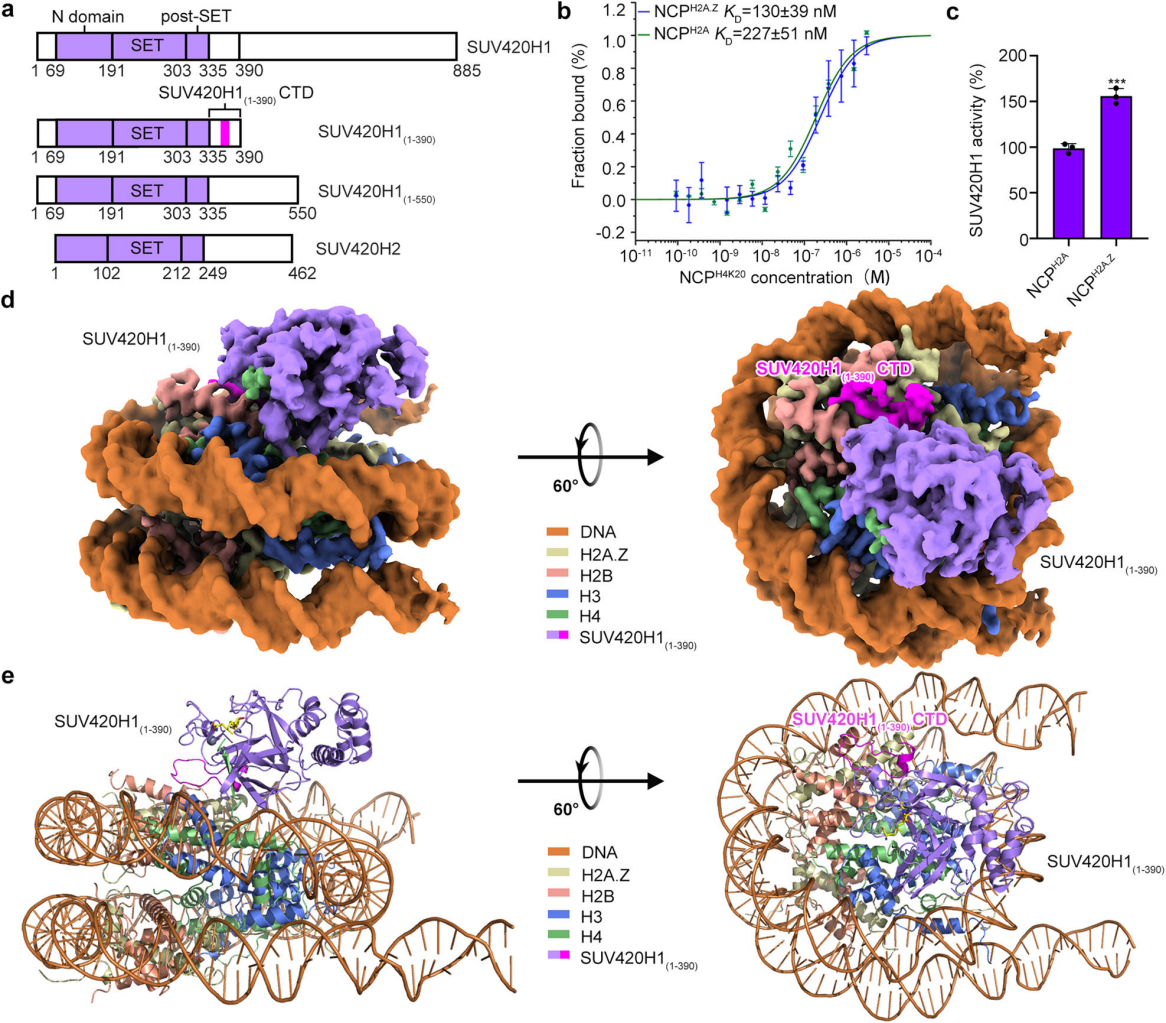
Biochemical analysis of SUV420H1 and the overall structure of the SUV420H1_(1–390)_–NCP^H2A.Z^ complex. **a** Schematic illustration of the domain structures of SUV420H1 and SUV420H2. Highlighted in the rectangle include the N-terminal domain, SET domain, post-SET domain, and SUV420H1_(1–390)_ CTD, and the region colored in magenta indicates the de novo modeling region. **b** MST assays of the binding of human SUV420H1_(1–390)_ to NCP^H2A^ and NCP^H2A.Z^. Dissociation constants (*K*_D_) = 227 ± 51 nM (NCP^H2A^) and 130 ± 39 nM (NCP^H2A.Z^). Error bars represent mean ± SEM based on three independent measurements. **c** Catalytic activity of the SUV420H1_(1–390)_ on NCP^H2A^ and NCP^H2A.Z^ by end-point HMT assays in vitro. Each assay was repeated at least 3 times with similar results. *n* = 3 independent experiments, two-tailed, unpaired *t*-test. ***P = 0.00086. **d**, **e** Cryo-EM density map (**d**) and atomic model (**e**) of human SUV420H1_(1–390)_–NCP^H2A.Z^ complex, shown in side (left) and top (right) view. The cryo-EM map is segmented and colored according to the components of the SUV420H1_(1–390)_–NCP^H2A.Z^ complex.

The expression of full-length human SUV420H1 in *Escherichia coli* cells turned out to be unstable for cryo-EM structural studies. Therefore, we screened SUV420H1 constructs with different truncations to improve SUV420H1 homogeneity. Finally, we found that the construct referred to as SUV420H1_(1__–__390)_ containing SUV420H1 residues 1–390 yielded high expression with ideal behavior in *E. coli* cells (Fig. 1a and Supplementary Fig. S1a). To stabilize the SUV420H1_(1–390)_ and NCPs complex for cryo-EM studies, we have introduced H4K20M mutation and found that SUV420H1_(1–390)_ bound to H4K20M NCPs (NCP^H4K20M^) more efficiency than canonical NCP^H4K20^ (Supplementary Fig. S1b, c). Microscale thermophoresis (MST)-based binding analysis showed that SUV420H1_(1–390)_ exhibits a binding affinity with a *K*_D_ value of 227 nM towards canonical NCP^H4K20^ (Supplementary Fig. S1b). In contrast, its binding affinity towards NCP^H4K20M^ increased by 3.7-fold, resulting in a *K*_D_ value of 62 nM (Supplementary Fig. S1b). Studies on H2A.Z facilitating the licensing and activation of replication origins revealed that H2A.Z-containing NCPs bind directly to the histone lysine methyltransferase SUV420H1, promoting H4K20me2 deposition^15^. As expected, MST-based binding analysis revealed that SUV420H1_(1–390)_ increased the binding affinity towards NCP^H2A.Z^ by a factor of 1.7-fold, with a *K*_D_ value of 130 nM (Fig. 1b), as compared to its binding to NCP^H2A^. The end-point histone methyltransferase (HMT) assay verified that the enzymatic activity of SUV420H1_(1–390)_ towards NCP^H2A.Z^ exhibited 1.6-fold as much as SUV420H1_(1–390)_ towards NCP^H2A^ (Fig. 1c). We then used Grafix^26^ method to further stabilize and purified the complex (Supplementary Fig. S1d) and determined the cryo-EM structures of SUV420H1_(1–390)_ in complex with H2A-containing NCPs (SUV420H1_(1–390)_–NCP^H2A^) and with H2A.Z-containing NCPs (SUV420H1_(1–390)_–NCP^H2A.Z^) at an overall resolution of 3.58 Å and 3.68 Å, respectively (Fig. 1d, e and Supplementary Figs. S2 and S3 and Table S1). In both cryo-EM structures, SUV420H1 made extensive contacts with the DNA and histones of NCPs. More importantly, we observed additional density corresponding to SUV420H1_(1–390)_ C-terminal domain (CTD) directly interacting with the H2A (H2A.Z)–H2B acidic patch (Fig. 1d, e).

### Nucleosome DNA and H4 tail recognition by SUV420H1

**Fig. 2:**
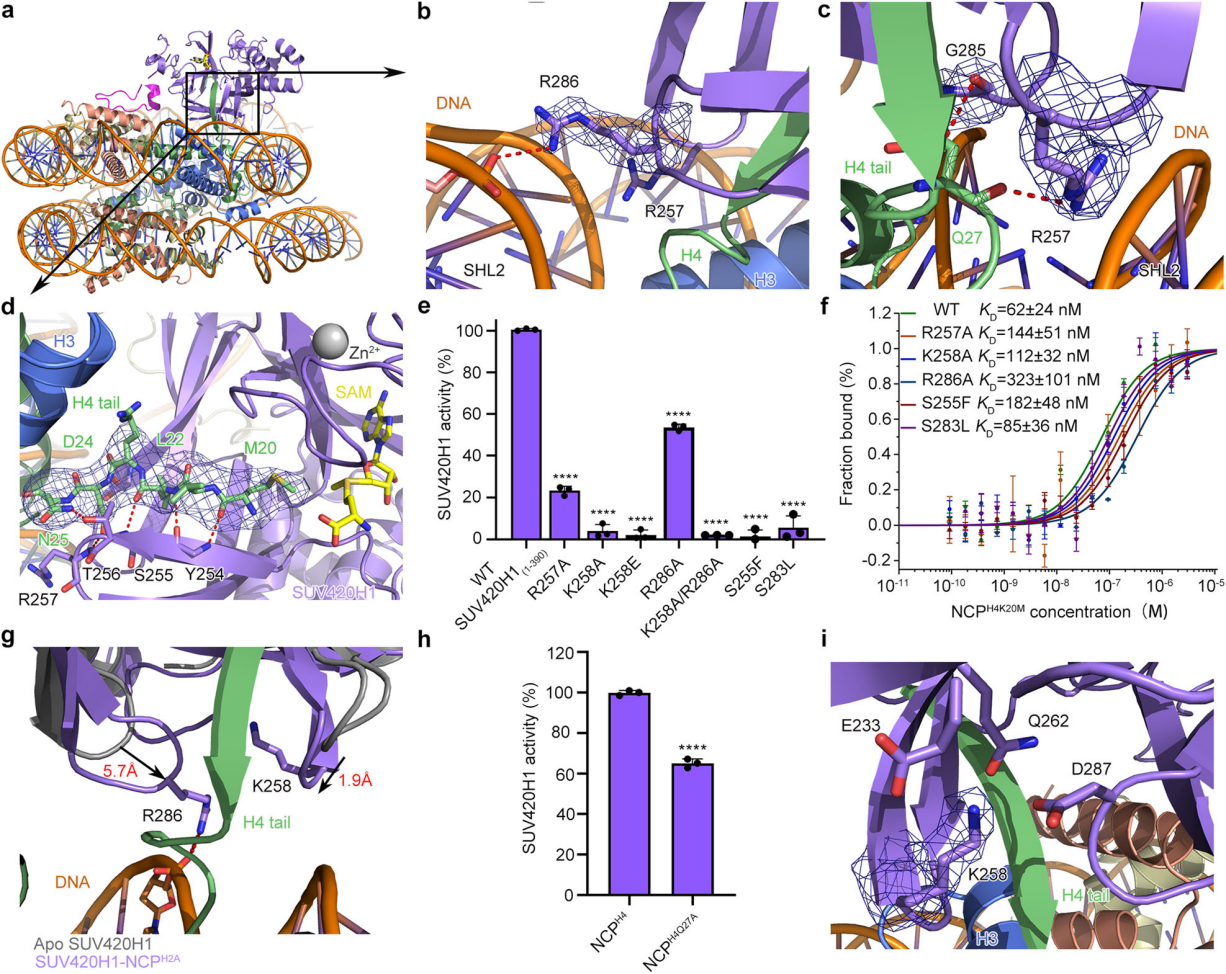
Interfaces between SUV420H1_(1–390)_ and the H4 tail together with DNA. **a** Overview of the recognition interfaces between SUV420H1_(1–390)_ and nucleosomal H4 tail and DNA components. SUV420H1_(1–390)_ domains are colored as shown in Fig. 1d. Histones H4 and DNA are colored in light green and orange, respectively. **b** Detailed view of the interactions between SUV420H1_(1–390)_ and the phosphate backbone of nucleosome SHL 2 DNA. Important residues at the interface are shown as sticks. **c** Detailed view of the interactions between SUV420H1_(1–390)_ and the H4 tail Q27. Important residues at the interface are shown as sticks. **d** Detailed view of the recognition interfaces between SUV420H1 and nucleosomal H4 tail. Residues at the interface of H4 are shown as sticks in green. H3 is colored in blue. SAM is colored in yellow. **e** Catalytic activity of wild-type SUV420H1_(1–390)_ and various mutants on NCP^H2A^ by end-point HMT assays in vitro. Adjusted *P* values for pairwise ANCOVA comparison of wild-type SUV420H1_(1–390)_ and each mutant are reported: **** *P* < 0.0001. **f** MST binding assays of wild-type SUV420H1_(1–390)_ and SUV420H1_(1–390)_ mutants on NCP^H2A^. Error bars represent mean ± SEM based on 3 independent measurements. **g** Alignment of the apo SUV420H1 (Protein Data Bank (PDB) code 3S8P, grey) with SUV420H1_(1–390)_–NCP^H2A^ complex (colored as in Fig. 1d). Directions of shifted regions of SUV420H1_(1–390)_–NCP^H2A^ are indicated with black arrows. **h** Catalytic activity of SUV420H1_(1–390)_ on NCP^H2A^ and NCP^H4Q27A^ by end-point HMT assays in vitro. ****P < 0.0001. **i** Detailed view of the interaction interface of SUV420H1 residue K258 in a negatively charged pocket. The important residues are shown as sticks.

In both structures, R286 of SUV420H1 recognizes the phosphate backbone of one strand spanning the DNA minor groove at superhelix location 2 (SHL 2) (Fig. 2a, b). R286 is a human cancer-associated mutational site^16^. To further consolidate its importance, we disrupted R286 with a mutation of R286A. MST binding assays showed that R286A decreases the binding affinity by 5.2-fold (*K*_D_ = 323 nM) towards NCP^H4K20M^ compared to wild-type SUV420H1_(1–390)_, and a loss of 46% catalytic activity towards nucleosome substrates (Fig. 2e, f and Supplementary Fig. S9), suggesting an important role of R286 in nucleosome recognition and H4K20 methylation.

The positively charged side chain of R257 is pointed towards the DNA minor groove through hydrogen bonds between the R257 side chain and the main chain of Q27 of H4. The side chain of H4 Q27 also forms a hydrogen bond with the main chain of G285 of SUV420H1 (Fig. 2c). The side chain of K258 inserts into a negatively charged pocket including residues E233, Q262, and D287 of SUV420H1 (Fig. 2i), further stabilizing the complex. Mutations of R257A and K258A result in decreased binding affinity to NCP^H4K20M^ by 2.3-fold and 1.8-fold, with *K*_D_ values of 144 nM and 112 nM (Fig. 2f), respectively, when compared to wild-type SUV420H1_(1–390)_. Notably, R257A mutant showed a 77% catalytic activity loss towards nucleosomal substrates (Fig. 2e and Supplementary Fig. S9). This implies that residue R257 plays a role in the catalytic activity of SUV420H1. Similarly, mutations of K258A, K258E and K258A/R286A double mutant nearly abolished the catalytic activity (Fig. 2e and Supplementary Fig. S9). The K258E mutation, which is found in uterine corpus endometrioid carcinoma^27^, indicates a loss of catalytic activity in patients harboring this K258E mutation. Furthermore, the mutation of Q27A in histone H4 leads to a 35% decrease in catalytic activity compared to wild-type SUV420H1_(1–390)_–NCP^H2A^ complex (Fig. 2h).

The interactions between the H4 tail and the *β* strand containing residues 252–256 of SUV420H1 are mainly mediated by hydrogen bonds between main-chain/side-chain atoms. Hydrogen bonds between T256 of SUV420H1 and N25, D24, L22 of H4, between Y254 of SUV420H1 and L22 of H4, between Y254 of SUV420H1 and M20 of H4 tightly position the side chain of M20 of H4 facing towards the methyl donor of the ligand S-adenosylmethionine (SAM) through insertion into the hydrophobic catalytic pocket of SUV420H1 (Fig. 2d and Supplementary Fig. S4a).

The S255F mutation is associated with breast-invasive carcinoma^27^, and the S283L mutation is identified in lung adenocarcinoma^28^. In our structure, we found that S255 and S283 are positioned at the entrance of that H4 tail insertion into the catalytic pocket (Supplementary Fig. S4b). When these serine residues are mutated to phenylalanine or leucine, the bulky side chain will block the entry for H4 tail insertion. Consequently, the binding of S255F to NCP^H4K20M^ was decreased by a factor of 2.9-fold, with a *K*_D_ value of 182 nM (Fig. 2f). Similarly, S283L resulted in a 1.4-fold reduction in binding to NCP^H4K20M^, with a *K*_D_ value of 85 nM (Fig. 2f). Furthermore, S255F and S283L demonstrated a substantial reduction in catalytic activity (Fig. 2e and Supplementary Fig. S9), which is consistent with findings from a previous study^20^. Compared with the apo structure of SUV420H1 (PDB 3S8P)^21^, the NCP-bound activated SUV420H1 catalytic loops adopted a more compact conformation. Specifically, residue R286 interacts with the nucleosomal DNA backbone and the side chain of Q27 from H4 tail forms hydrogen bond with the main chain of G285 of SUV420H1 (Fig. 2b, c), causing the loop containing residues 283–288 of SUV420H1 undergoing a movement of ~5.7 Å towards the H4 tail (Fig. 2g). This conformational change suggests the involvement of the loop containing residues 283–288 in the catalytic activity of SUV420H1. Similarly, the loop containing residue K258 moves around 1.9 Å towards the H4 tail. This movement indicates that K258 plays a role in the interaction with the H4 tail and may contribute to the catalytic activity of SUV420H1 when bound to the nucleosome core particles (Fig. 2g), further facilitating SUV420H1 recognition of nucleosome DNA and H4 tail. These observed conformational changes in the catalytic loops highlight their dynamic nature and their importance in mediating the recognition and binding of the H4 tail by SUV420H1 when it is in a complex with nucleosomes. Interestingly, the structure of NCP-bound SUV420H1–H4 resembles the crystal structure of SUV420H2–H4 complex (PDB 4AU7), with the difference that the DNA interacting loops get closer to the nucleosomal DNA (Supplementary Fig. S4c).

### SUV420H1_(1–390)_ CTD recognizes H2A (H2A.Z)–H2B acidic patch

**Fig. 3:**
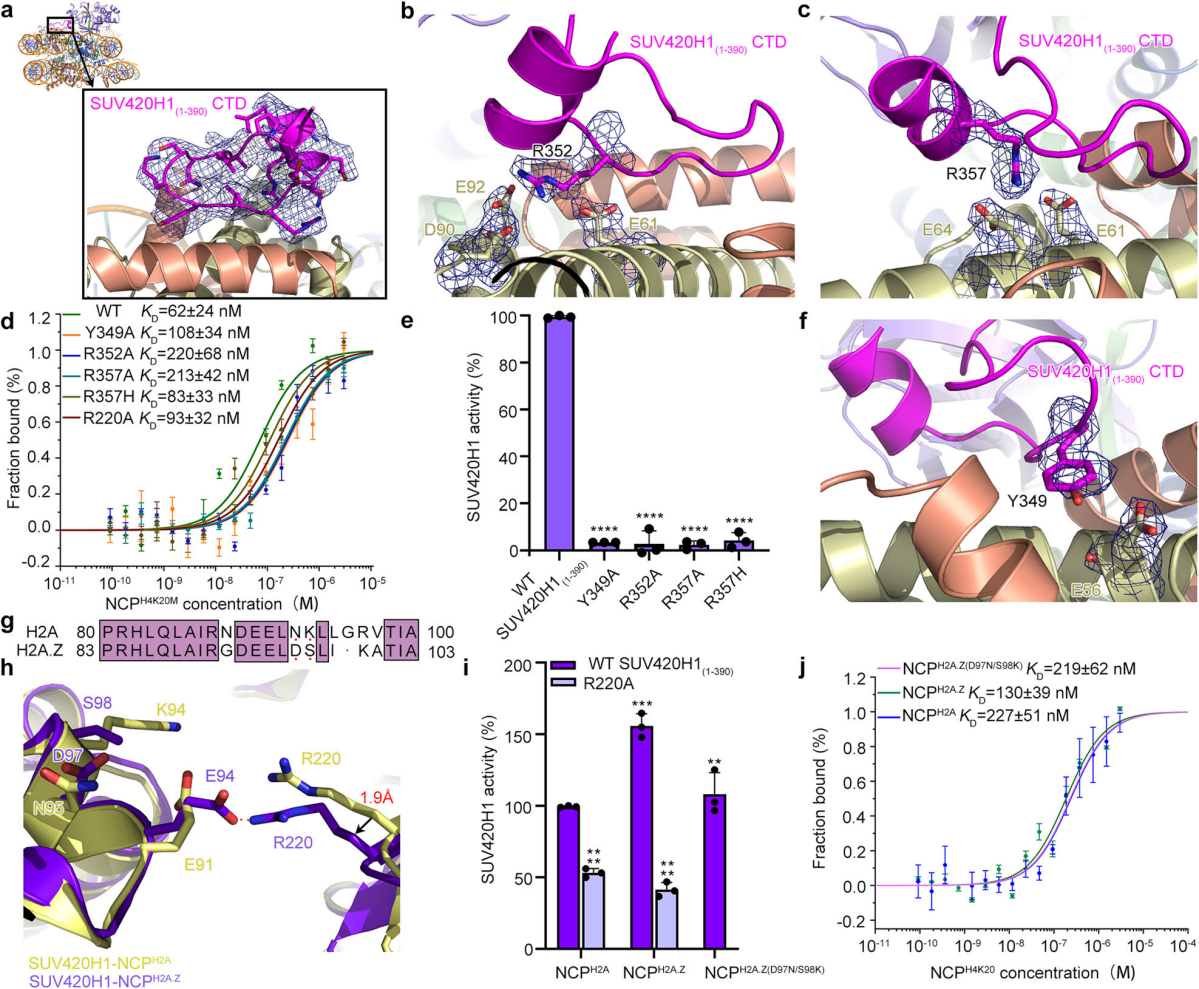
Contacts with H2A–H2B acidic patch by SUV420H1_(1–390)_ CTD domain orient SUV420H1 onto nucleosome. **a** Overview of the contacts between SUV420H1_(1–390)_ CTD domain and nucleosomal H2A–H2B acidic patch. SUV420H1_(1–390)_ are colored the same as Fig. 1d. Histones H2A and H2B are colored in yellow and pink, respectively. **b**, **c**, **f** Detailed view of the recognition of the acidic patch of the nucleosome by arginine anchors. Important residues at the interface are shown as sticks. **d** MST binding assays of wild-type SUV420H1_(1–390)_ and SUV420H1_(1–390)_ mutants with NCP^H4K20M^. Error bars represent mean ± SEM based on three independent measurements. **e** Catalytic activity of wild-type SUV420H1_(1–390)_ and various mutants on NCP^H2A^ by end-point HMT assays in vitro. Each assay was repeated at least three times with similar results. ****P < 0.0001. **g** Sequence alignment of the region containing D97 and S98 of H2A.Z and the corresponding region of H2A (in single-letter code). The same residues are boxed in a purple background. **h** Conformational changes and shifts of residue 220 in SUV420H1_(1–390)_–NCP^H2A^ and SUV420H1_(1–390)_–NCP^H2A.^^Z^ complexes. Directions of shifted regions are indicated with black arrows. **i** Catalytic activity of SUV420H1_(1–390)_ on NCP^H2A^, NCP^H2A.Z^ and NCP^H2A.Z^^(D97N/S98K)^ by end-point HMT assays in vitro. The catalytic activity of SUV420H1_(1–390)_ R220A on NCP^H2A^ and NCP^H2A.Z^ by end-point HMT assays in vitro. Each assay was repeated at least three times with similar results. ***P = 0.0006, **P = 0.0086. **j** MST binding assays of wild-type SUV420H1_(1–390)_ on NCP^H2A^, NCP^H2A.Z^, NCP^H2A.Z(D97N/S98K)^ containing H4K20.

Interestingly, in both of the cryo-EM density maps of SUV420H1_(1–390)_–NCP^H2A^ and SUV420H1_(1–390)_–NCP^H2A.Z^ complexes, we observed additional SUV420H1 density interacting with nucleosome H2A (H2A.Z)–H2B acidic patch that is good enough to be modeled. The residues 341–361 of SUV420H1 were built into this region by de novo modeling, assisted with the predicted models from AlphaFold Protein Structure Database^29^. Recognition of the acidic patch of the nucleosome by arginine anchors^30^ is widely found in histone lysine methyltransferases, including Dot1L, Set8, and COMPASS^24,31,32^. The structure reveals that two arginine anchors of the SUV420H1_(1–390)_ CTD make extensive interactions with the acidic patch of the nucleosome. The side chain of the positively charged residue R352 inserts into a negatively charged network that is composed of residues E61, D90, and E92 of H2A (corresponding to residues E64, D93 and E95 of H2A.Z), enabling SUV420H1 to engage with nucleosome by electrostatic interactions (Fig. 3a, b). R357 is positioned close to the negatively charged residues E61 and E64 of H2A (corresponding to residues E64 and E67 of H2A.Z), forming electrostatic interactions (Fig. 3c). In addition to the arginine anchors, residue Y349 forms hydrogen bond with E56 in H2A (corresponding to residue E59 of H2A.Z) (Fig. 3f). To investigate the functions of Y349, R352 and R357, we reconstituted Y349A, R352A, R357A mutations and measured the binding affinity and catalytic activity of the mutant proteins towards NCP^H2A^. As expected, Y349A, R352A and R357A displayed reductions in binding affinity towards NCP^H2A^ substrates, with respective decreases of 1.7-fold, 3.5-fold and 3.4-fold. Their *K*_D_ values were measured at 108 nM, 220 nM and 213 nM, respectively, in comparison with wild-type SUV420H1_(1–390)_ with a *K*_D_ value of 62 nM (Fig. 3d). Moreover, the catalytic activities of Y349A, R352A and R357A mutants towards NCP^H2A^ were nearly abolished in vitro (Fig. 3e and Supplementary Fig. S9), when compared with the wild-type SUV420H1_(1–390)_, suggesting that the interactions between residues Y349, R352 and R357 of SUV420H1 and the acidic patch of nucleosomes are necessary for SUV420H1 to exhibit full methylation activity on nucleosomes. Notably, the recurrent cancer-associated mutation R357H^16^ led to a slight reduction in binding towards NCP^H2A^, with a *K*_D_ value of 83 nM. However, the catalytic activity of R357H mutation towards NCP^H2A^ was almost lost (Fig. 3e and Supplementary Fig. S9), in support of the functional importance of the arginine anchors of SUV420H1 for maximal activity. The two arginine anchors (R352, R357) and Y349 of SUV420H1 are highly conserved in SUV4-20H family proteins (Supplementary Fig. S5), illustrating a common mechanism for nucleosome acidic patch recognition by SUV4-20H family enzymes.

H2A.Z has been previously reported to enhance the binding of SUV420H1, thereby promoting its enzymatic activity^15^, but this mechanism remains poorly understood. The different acidic patch residues D97/S98 in H2A.Z corresponding to N94/K95 in H2A are important in nucleosomal surface alteration further affecting chromatin factors binding (Fig. 3g)^33^. We generated double mutant D97N/S98K of H2A.Z and measured the binding affinity and catalytic activity of SUV420H1 (Fig. 3i, j). SUV420H1 exhibited similar catalytic activity and binding affinity on H2A.Z D97N/S98K NCP as it did on canonical NCP^H2A^ (Fig. 3i, j), which demonstrates that residues D97 and S98 of H2A.Z play a predominant role in enhancing the binding and catalytic activity of SUV420H1 towards NCP^H2A.Z^, consistent with a previous study^15^. To explore whether SUV420H1 has specific features enabling enhanced binding or catalytic activity on H2A.Z nucleosomes, we aligned SUV420H1–NCP^H2A^ complex with SUV420H1–NCP^H2A.Z^ complex, the two structures superimposed well with each other, except for some minor differences. Interestingly, we observed that residue R220 of SUV420H1 pointing to H2A/H2A.Z acidic patch undergoes slight conformational change, such that the distance between R220 and E94 of H2A.Z is closer than the distance between R220 and E91 of H2A (Fig. 3h and Supplementary Fig. S6a–c). To further investigate the role of R220 in SUV420H1 methylation, we introduced a mutation, R220A, into SUV420H1_(1–390)_ and measured the binding affinity and catalytic activity. In comparison to the wild-type SUV420H1_(1–390)_, R220A mutant displayed a decreased binding affinity toward NCP^H2A^, with a 1.5-fold reduction and a *K*_D_ value of 93 nM (Fig. 3d). In the end-point HMT assay, the catalytic activity of R220A mutant towards NCP^H2A^ decreased by 1.9-fold when compared to wild-type SUV420H1_(1–390)_ (Fig. 3i). Additionally, the catalytic activity of R220A mutant towards NCP^H2A.Z^ decreased by 3.8-fold in comparison to wild-type SUV420H1_(1–390)_ (Fig. 3i and Supplementary Fig. S9), suggesting that R220 of SUV420H1 plays a role in regulating the enhanced activity of SUV420H1 on H2A.Z nucleosomes.

**Fig. 4:**
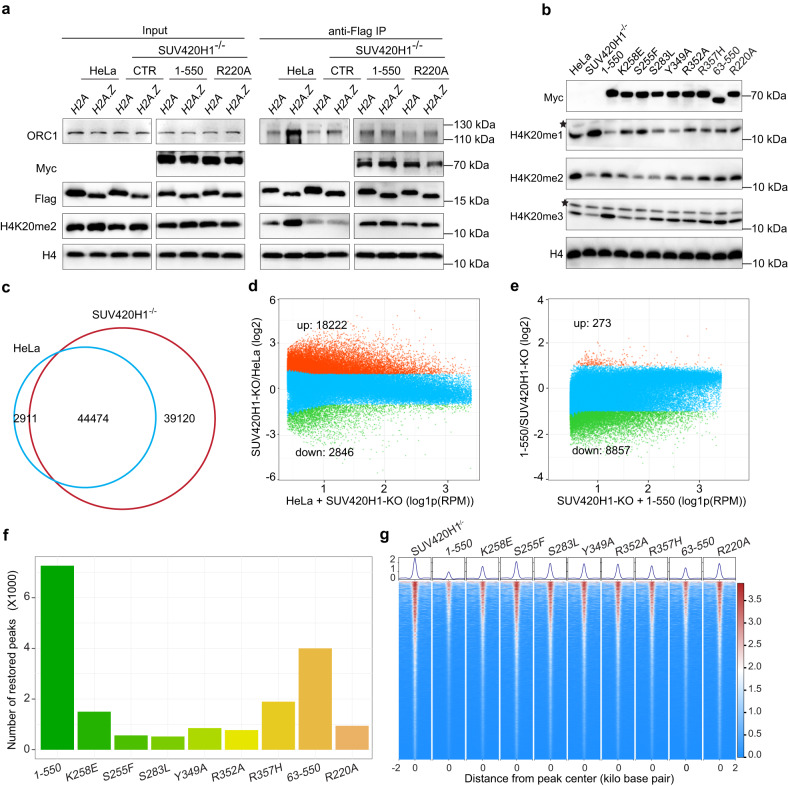
Functional validation of SUV420H1–NCP complex. **a** Western blot assay shows the enrichment of H4K20me2, SUV420H1 and ORC1 on Flag-H2A or Flag-H2A.Z nucleosomes, after mono-nucleosome IP. Mono-nucleosome IP was performed in wild-type HeLa cells, SUV420H1^–/–^ HeLa cells and SUV420H1^–/–^ HeLa cells ectopically expressing Myc-SUV420H1_(1–550)_ or Myc-SUV420H1_(1–550)_-R220A mutant. **b** Western blot assay shows the levels of H3K4me1/2/3 after over-expressing SUV420H1 _(1–550)_ or SUV420H1 _(1–550)_ with a single mutation of K258E, S255F, S283L, Y349A, R352A, R357H or R220A in SUV420H1^–/–^ HeLa cells, whose expression is indicated by Myc. The stars indicate a non-specific signal from H4K20me1 or H4K20me3 antibody. **c** Venn plot shows the overlapping of ATAC-seq peaks from wild-type and SUV420H1^–/–^ HeLa cells. **d** Dot plot shows the dynamics of ATAC-seq signal in wild-type and SUV420H1^–/–^ HeLa cells within the ATAC-seq peaks (*n* = 75,999) from SUV420H1^–/–^ HeLa cells. Up (*n* = 18,222) and down (*n* = 2846) indicate peaks with increased or decreased ATAC-seq signal filtered with two-fold changes and *P* < 0.01. **e** Dot plot shows the dynamic changes of ATAC-seq signal after over-expressing SUV420H1_(1–550)_ in SUV420H1^–/–^ HeLa cells, within the ATAC-seq peaks (*n* = 75,999) from SUV420H1^–/–^ HeLa cells. Up (*n* = 273) and down (*n* = 8857) indicate peaks with increased or decreased ATAC-seq signal filtered with two-fold changes and *P* < 0.01. **f** Bar plot shows the number of peaks with decreased ATAC-seq signal after over-expressing SUV420H1 _(1–550)_ or SUV420H1 _(1–550)_ with a single mutation of K258E, S255F, S283L, Y349A, R352A, R357H or R220A, filtered with two-fold changes and *P* < 0.01. **g** Heatmap shows the ATAC-seq signal around the center of the 8857 “down” ATAC-seq peaks as indicated in **e**, from SUV420H1^–/–^ HeLa cells or SUV420H1^–/–^ HeLa cells over-expressing SUV420H1_(1–550)_ or SUV420H1 _(1–550)_ with a single mutation as indicated in **f**.

To validate the function of R220 of SUV420H1, we ectopically expressed residues 1–550 of SUV420H1 (hereafter named SUV420H1_(1–550)_) (Fig. 1a) or SUV420H1_(1–550)_-R220A mutant together with Flag-H2A or Flag-H2A.Z histones in a HeLa SUV420H1^–/–^ cell line. Then we performed mono-nucleosome immunoprecipitation (IP) using anti-Flag-agarose beads. We observed that H4K20me2 and ORC1 are enriched on H2A.Z nucleosomes compared to H2A nucleosomes, and this enrichment is dependent on SUV420H1 (Fig. 4a). These findings align with the study by Long et al.^15^. After ectopically expressing wild-type SUV420H1_(1–550)_ in HeLa SUV420H1^–/–^ cells, we found that SUV420H1_(1–550)_ exhibits a stronger binding on H2A.Z nucleosomes than on H2A nucleosomes (Fig. 4a), which is consistent with the results of MST binding assays in vitro (Fig. 3j). Whereas, the binding of SUV420H1_(1–550)_-R220A mutant to H2A.Z nucleosomes is reduced to a lower degree than to H2A nucleosomes (Fig. 4a), and this is consistent with the more reduction of catalytic activity of SUV420H1_(1–390)_-R220A mutant on NCP^H2A.Z^ than on NCP^H2A^ (Fig. 3i). However, even SUV420H1_(1–550)_-R220A mutant cannot restore H4K20me2 and ORC1 as efficiently as wild-type SUV420H1_(1–550)_ did, the enrichment of H4K20me2 and ORC1 on H2A.Z nucleosomes relative to H2A nucleosomes was still retained (Fig. 4a). Taken together, these results suggested that while R220 plays a role in enhancing SUV420H1’s binding activity on H2A.Z nucleosomes, and regulating SUV420H1’s catalytic activity, it cannot fully explain SUV420H1’s enhanced activity on H2A.Z nucleosomes relative to H2A nucleosomes.

### Functional validation of SUV420H1–NCP complex

As we have observed multiple interactions between SUV420H1 and nucleosome that are critical for the binding and catalytic activity of SUV420H1 towards nucleosome substrate, we further tested the role of these interactions under physiological conditions. We ectopically expressed SUV420H1_(1–550)_ or SUV420H1_(1–550)_ with a single K258E, S255F, S283L, Y349A, R352A, R357H or R220A mutation in a HeLa SUV420H1^–/–^ cell line. After knocking out SUV420H1, we observed a strong reduction of H4K20me2 and to a lesser extent reduction of H4K20me3, and in parallel an increase of H4K20me1 (Fig. 4b), which is consistent with a previous report^12^. Moreover, we observed that the defects of H4K20me2/3 are mostly rescued by ectopically expressing SUV420H1_(1–550)_ (Fig. 4b). Thus, the dynamics of H4K20me1/2/3 we observed is specific to SUV420H1 in the HeLa SUV420H1^–/–^ cell line. We found that compared to SUV420H1_(1–550)_, mutants S255F and S283L show no rescue effect on both H4K20me2 and H4K20me3 (Fig. 4b), which is consistent with the critical role of S255 and S283 in the catalytic core of SUV420H1. We also found that mutants K258E, Y349A, and R352A only show a partial rescue effect on H4K20me2, and even less on H4K20me3 (Fig. 4b). Mutants R357H and R220A restored H4K20me2 almost as SUV420H1_(1–550)_ did, but it cannot fully rescue H4K20me3 (Fig. 4b). These results supported that residues K258, Y349, R352, R357, and R220 play roles in facilitating the binding and catalytic activity on nucleosome substrate under physiological condition. We also noted that deletion of residues 1–62 of SUV420H1_(1–550)_ did not impair its catalytic activity obviously (Fig. 4b), which is consistent with in vitro HMT assay (Supplementary Figs. S8c and S9). It has been reported that H4K20me2/3 is involved in the regulation of heterochromatin^10^. Consistently, through Assay for Transposase Accessible Chromatin using sequencing (ATAC-seq) we found that the number of open regions increased dramatically after SUV420H1 knockout (Fig. 4c). Then we quantified the dynamics of the ATAC-seq signal in the peak regions. We found that 18,222 regions are more open in SUV420H1^–/–^ cells, while only 2846 regions are less open (Fig. 4d). Importantly, after ectopically expressing SUV420H1_(1–550)_ in SUV420H1^–/–^ cells, 8857 (11.9%) of the peak regions significantly (*P* < 0.01) decreased ATAC-seq signal by 2-fold (Fig. 4e), suggesting that these chromatin regions are restored to closed state. Through the same criteria, we analyzed the chromatin states by ATAC-seq after ectopically over-expressing SUV420H1_(1–550)_ mutants in HeLa SUV420H1^–/–^ cells. We found that, after over-expressing K258E, S255F, S283L, Y349A, R352A, R357H or R220A mutants, the number of peaks showing decreased ATAC-seq signal is less than that after over-expressing SUV420H1_(1–550)_ (Fig. 4f), indicating that none of the mutants can restore the chromatin states as efficiently as SUV420H1_(1–550)_ does. Through analyzing ATAC-seq signal in the 8857 “down” peaks as indicated in Fig. 4d, we found that the ATAC-seq signal decreased much less when over-expressing the mutants than over-expressing SUV420H1_(1–550)_ (Fig. 4g). These results supported that K258E, S255F, S283L, Y349A, R352A, R357H and R220A mutation impaired the activity of SUV420H1 in restoring the chromatin states, which is consistent with the defects of these mutants in rescuing H4K20me2/3.

## Discussion

The direct interaction between the SUV420H1 and nucleosome demonstrates that SUV420H1 recognizes three elements, the H4 tail, the acidic patch, and the DNA component to achieve a tight binding like other histone methyltransferases^24,32,34^. It is observed in our structure that the breast-invasive carcinoma and lung adenocarcinoma cancer-associated mutations S255F^27^ and S283L^28^ with larger sidechains block the insertion of the H4 tail into SUV420H1 SET domain at the entrance site (Supplementary Fig. S4b), thus inhibiting the methylation activity of SUV420H1 confirmed by in vitro and in vivo assays^20^. For cancer-associated mutation K258E^27^, it disrupts the interaction of K258 with a negatively charged pocket (Fig. 2i). This disruption prevents the stabilization of the SUV420H1 complex bound to the nucleosome, leading to a loss of function. SUV420H1 anchors on nucleosome through R357 for its full activity. The cancer-related mutant R357H^16^ impairs the methylation activity of SUV420H1 in vitro and cannot fully rescue H4K20me3 in SUV420H1 in HeLa SUV420H1^–/–^ cells. However, as SUV420H2 can also catalyze H4K20me3^12^, the deficiency of SUV420H1 mutants in rescuing H4K20me3 may have been partially masked. Overall, these findings provide structural evidence for how SUV420H1 recognizes and interacts with nucleosomes, and they elucidate the mechanisms through which disease-associated mutations in SUV420H1 inhibit its methylation activity in the context of nucleosomes.

Our results demonstrated that the enzymatic activity of SUV420H1_(1–390)_ towards NCP^H2A.Z^ was 1.6-fold higher compared to SUV420H1_(1–390)_ towards NCP^H2A^ (Fig. 1b) as observed in the end-point HMT assay. In previous reports, the difference in methylation activity between SUV420H1 on NCP^H2A^ and NCP^H2A.Z^ was even more significant^15^, as they used ^3^H labeling SAM methyl group methods. The variations in measured activity may be attributed to the use of different detection methods. Based on our observation, we studied the role of R220 of SUV420H1 in enhancing the binding and catalytic activity on H2A.Z nucleosomes. As we observed in our structure, R220 positioned closer to E94 of H2A.Z than it with E91 of H2A (Fig. 3h), thus it may enhance SUV420H1 binding on H2A.Z nucleosomes through electronic interaction between R220 and E94 of H2A.Z. However, R220 is not directly located within the catalytic pocket, suggesting that there might be other mechanisms in regulating SUV420H1’s activity on H2A.Z nucleosomes other than the preferential binding.

In our research, we observed a minor degree of DNA unwrapping in SUV420H1_(1–390)_–NCP^H2A.Z^ complex structure compared to the H2A nucleosome (PDB 7KTQ) (Supplementary Fig. S7b), while there was no apparent DNA unwrapping in SUV420H1_(1–390)_–NCP^H2A^ complex structure (Supplementary Fig. S7a). A previous report has indicated that the DNA termini in H2A.Z nucleosomes are more flexible compared to H2A nucleosomes^35^ (Supplementary Fig. S7c). Notably, the level of unwrapping in the SUV420H1_(1–390)_–NCP^H2A.Z^ complex closely resembles that observed in H2A.Z nucleosomes (Supplementary Fig. S7d). The binding of SUV420H1 is enhanced by NCP^H2A.Z^, which promotes its enzymatic activity^15^. Our observations suggest that DNA unwrapping at the termini within the SUV420H1_(1–390)_–NCP^H2A.Z^ complex could be influenced by the inherent unwinding properties of NCP^H2A.Z^ DNA. This flexibility in the unwrapped DNA termini in H2A.Z nucleosomes might potentially facilitate easier access for SUV420H1, leading to the enhancement of SUV420H1’s catalytic activity. However, further investigations are warranted to this phenomenon.

It has been reported that H4K20me2/3 are deposited at pericentric heterochromatin^9^ or telomeric heterochromatin^36^ by SUV420H1/2, which is recruited to these regions via Heterochromatin Protein 1 (HP1) in an H3K9me3 dependent manner^9^. In skeletal muscle stem cells (MuSCs), SUV420H1 promotes the formation of facultative heterochromatin to regulate MuSC quiescence^37^. However, whether and how H4K20me2/3 is directly involved in the regulation of heterochromatin at genome-wide is not clear. In our study, we found by ATAC-seq, which is widely used to map open chromatin regions, that after knockout of SUV420H1 in HeLa cells, the chromatins transit into a hyper-open state, suggesting that SUV420H1 mediated H4K20me2/3 may also play a role in coordinating the activity of *cis*-regulatory elements. In concordance with this speculation, SUV420H1-catalyzed H4K20me2 can recruit ORC1 to facilitate the activation of replication origins^6,15^. Importantly, we identified critical interactions within the SUV420H1_(1–390)_–NCP^H2A.Z^ complex that is required for the full activity for SUV420H1, which may be useful to dissect the role of H4K20me2/3 in the regulation of heterochromatin without disrupting SUV420H1 entirely.

We observed an additional density in our cryo-EM density map that could not be attributed to a specific atom model, possibly corresponding to the N-terminal region of SUV420H1 (Supplementary Fig. S8a). This observation led us to further investigate the binding affinity and enzymatic activity of different SUV420H1 truncations. Interestingly, we found that SUV420H1_(1–335)_ (*K*_D_ = 292 nM), exhibited a 4.7-fold lower binding affinity to nucleosomes compared to SUV420H1_(1–390)_ (*K*_D_ = 62 nM). However, the truncation residues 63–390 of SUV420H1 (hereafter named SUV420H1_(63–390)_), which lacks residues 1–62 of the N-terminal region, displayed a 6.6-fold decrease in binding affinity (*K*_D_ = 410 nM) compared to SUV420H1_(1–390)_ (Supplementary Fig. S8b). Despite this decrease in binding affinity, SUV420H1_(63–390)_ demonstrated comparable enzymatic activity to SUV420H1_(1–390)_, whereas the catalytic activity of SUV420H1_(1–335)_ was almost abolished (Supplementary Figs. S8c and S9). This indicates that the deletion of residues 1–62 primarily affects the binding affinity of SUV420H1 to nucleosomes without impairing its methylation activity. Additionally, our results suggest that the CTD of SUV420H1 is essential for its methylation activity and binding affinity towards nucleosome substrates.

Overall, our findings provide valuable structural insights into the mechanisms of nucleosome recognition and methylation by SUV420H1. Furthermore, we identified key residues and interaction interfaces that contribute to the enhanced activity of SUV420H1 towards nucleosomes containing the variant histone H2A.Z compared to canonical nucleosomes. These structural elements and interactions uncovered in our study serve as important targets for further functional investigations of SUV420H1 and its role in chromatin regulation.

## Materials and methods

### Protein expression and purification

Human SUV420H1 protein and its truncations or mutants were cloned into a modified pET28a vector with an N-terminal 6× His-TEV tag. The expression and purification of SUV420H1 and mutants were performed using the same protocol. In general, SUV420H1 was transformed into *E. coli* BL21(DE3)-RIL competent cells and were induced with 0.4 mM IPTG at 18 °C for an overnight expression. The cells were centrifuged at 5000 rpm for 15 min at 4 °C, resuspended in lysis buffer (25 mM HEPES, pH 7.5, 500 mM NaCl, 5% glycerol, 1 mM TCEP) and then sonicated for around 30 min. The supernatant was collected by centrifugation of the cell lysate at 20,000 rpm for 1 h and was added to Ni-NTA resin (BOGELONG) that had been equilibrated in buffer A (25 mM HEPES, pH 7.5, 500 mM NaCl, 5% glycerol, 1 mM TCEP). The beads were incubated with the lysate at 4 °C for 1 h, poured into a gravity flow column and washed with buffer A. Protein was eluted using buffer B (25 mM HEPES, pH 7.5, 500 mM NaCl, 5% glycerol, 1 mM TCEP, 300 mM imidazole). After elution 1 mg of TEV protease was added directly to the eluate and dialyzed overnight at 4 °C against SUV420H1 dialysis buffer (25 mM HEPES, pH 7.5, 500 mM NaCl, 5% glycerol, 1 mM DTT). Precipitate was removed by centrifugation and filtration through an Amicon Ultra spin concentrator (Millipore). To separate SUV420H1 from TEV and the cleaved 6× His-tag, the sample was again loaded onto the Ni-NTA resin equilibrated in buffer A, collecting the flow through. The protein was concentrated and was further purified by a Superdex increase 200 16/600 size-exclusion chromatography column (GE Healthcare) that was pre-equilibrated with SEC buffer (25 mM HEPES, pH 7.5, 300 mM NaCl, 5% glycerol, 1 mM DTT). Peak fractions corresponding to SUV420H1 were pooled and the pure concentrated protein (15 mg/mL) was stored at –80 °C freezer until use.

### Nucleosome reconstitution

Histone H2A, H2A.Z, H2B, H3.1, H4 were cloned into pET22b vector. H4K20M was generated using site-mutagenesis introduced by primers. The expression and purification of histones were performed using the same protocol. Briefly, the histone plasmid was transformed into *E. coli* BL21(DE3) and expressed in an insoluble form at 37 °C. The cells were lysed and the insoluble bodies were collected, and dialyzed in the denaturing buffer (20 mM sodium acetate pH 5.2, 7 M urea, 0.2–1 M NaCl, 1 mM EDTA, 5 mM 2-mercaptoethanol). After being purified by an SP sepharose HP (GE Healthcare) column, the histone proteins were dialyzed completely against distilled water containing 2 mM β-mercaptoethanol, and were concentrated to 2 mg/mL and then lyophilized to store at –80 °C.

The plasmid containing 12 × 167-bp 601 DNA fragment^38^ was constructed using *E. coli* DH5α strain. The pUC19-12 × 167 plasmid was large-scale purified and the 167-bp fragment was excised from plasmid by *Eco*RV digestion and isolated from the digestive product as described^39^.

Nucleosome core particles were reconstituted as described previously^40,41^. In brief, histone octamers were prepared by mixing H2A, H2B, H3.1, and H4 in a refolding buffer containing 2 M NaCl. Histone octamers were isolated by size-exclusion chromatography, concentrated, and stored at –80 °C. Then, the purified histone octamer was mixed with the 167-bp 601 DNA at a molar ratio of 1:1.1 and was dialyzed against reconstitution buffer (10 mM Tris-HCl, pH 7.5, 1 mM EDTA, 1 mM DTT, 0.25–2 M KCl) by salt gradient dilution for 36 h. The reconstituted NCP was further purified through superdex increase 200 16/600 gel filtration, and was then stored at 4 °C.

### Electrophoretic mobility shift assay (EMSA)

SUV420H1 was mixed with 1 pmol NCP^H2A^ or NCP^H2A.Z^ at a molar ratio of 0:1, 3:1 and 9:1 to a total volume of 10 μL. After incubation on ice for 30 min in EMSA buffer (25 mM HEPES, pH 7.5, 50 mM NaCl, 50 mM KCl, 2 mM DTT, 40 μM SAM), the samples were loaded onto a 6% native TBE gel at 4 °C. Electrophoresis was performed at 4 °C for 90 min at a constant voltage of 120 V. The resulting gels were visualized by GelRed staining and visualized.

### Assembly of the SUV420H1–NCP complexes

Wild-type SUV420H1 was mixed with nucleosome containing H2A or H2A.Z at a molar ratio of 10:1 in binding buffer containing 50 mM KCl, 50 mM NaCl, 25 mM HEPES, pH 7.5, 40 μM SAM, and 2 mM DTT at 4 °C for 15 min.

The above samples were then subjected to GraFix^26^. Five hundred microliters of sample were put into the 12.5-mL tubes with a 10%–30% linear glycerol gradient and a 0–0.025% linear glutaraldehyde gradient in 25 mM HEPES, pH 7.5, 100 mM NaCl and 1 mM DTT, then centrifugated at 35,000 rpm for 16 h using a Beckman SW41Ti rotor at 4 °C. Following centrifugation, the sample was divided into 200 μL each fraction from top to bottom, and the fractions were examined by 6% native TBE gel and negative-staining EM. Fractions containing uniform, properly sized particles were pooled and centrifugated to remove glycerol and concentrate.

### Cryo-EM sample preparation and data collection

For the cryo-EM specimen preparation of both the SUV420H1_(1–390)_–NCP^H2A^ and the SUV420H1_(1–390)_–NCP^H2A.Z^ complexes, Quantifoil R1.2/1.3 200 mesh Au grids were glow-discharged for 40 s using a Gatan Plasma System. With the Vitrobot (ThermoFisher Scientific) set to 8 °C, 100% humidity, 3 μL of the samples were loaded onto the grid waiting for 10 s, and then blotted for 3.0 s, directly plunged into liquid ethane.

Micrographs were collected on a Titan Krios microscope, operated at 300 kV, equipped with a K3 Summit direct electron detector (Gatan). SerialEM software (ThermoFisher Scientific) was used for automated data collection^42^ in super-resolution mode at a nominal magnification of ×130,000, corresponding to a calibrated pixel size of 0.92 Å at the object scale. The defocus range was set from –1 to –2 μm. Each micrograph was dose-fractioned to 32 frames at a dose rate of 1.5652 e^−^/pixel/s, with a total exposure time of 2.1 s, resulting in a total dose of about 50 e^−^/Å^2^.

### Image processing

Both the SUV420H1_(1–390)_–NCP^H2A^ and SUV420H1_(1–390)_–NCP^H2A.Z^ data sets were processed in RELION 3.0^43^ and CryoSPARC^44^. Motion correction and dose-weighted motion correction were performed using MotionCor2^45^. Gctf was used for CTF parameter estimation^46^. For the SUV420H1_(1–390)_–NCP^H2A^ data set (4106 micrographs), 1,201,595 particles were automatically picked and extracted in RELION. The autopicked particles were subjected to 4 rounds of 2D classifications. Then, 873,129 selected particles from the last 2D classification were subjected to 3D classification. The class of 158,987 particles with good SUV420H1 density was selected and re-extracted for a round of refinement. After that, to obtain better SUV420H1 density, local classification procedures were performed with the “--skip_alignment” option in RELION, and a few critical parameters, such as the mask size and regularization parameter T, were extensively tested. Finally, a total of 35,448 particles were selected for further refinement and postprocessing, yielding final reconstructions at overall resolution of 3.68 Å. The final selected good particles were transferred to CryoSPARC for a second refinement with some regions with better density.

For the SUV420H1_(1–390)_–NCP^H2A.Z^ data set (6794 micrographs), the processing procedures were the same. 1,782,793 particles were automatically picked and subjected to 3 rounds of 2D classifications in RELION. The selected 984,971 particles were subjected to two rounds of 3D classifications. Then 308,576 particles were selected and re-extracted for a round of refinement. Local classification procedures were performed with the “--skip_alignment” option in RELION, with the mask size and regularization parameter T tested. Then 45,377 particles were selected for final refinement and postprocessing, yielding an overall resolution of 3.68 Å. The final selected good particles were transferred to CryoSPARC for refinement and get a map with better SUV420H1 density.

### Model building, refinement and validation

Cryo-EM structure of NCP complex (PDB 5WCU)^47^ and crystal structures of the SET domain of human SUV420H1 (PDB 3S8P)^21^ were used as initial models. These initial models were docked into the cryo-EM density maps in Chimera^48^ and manually adjusted in Coot^49^. The residues 341–361 of SUV420H1 were manually built de novo in Coot with the help of the predicted models from AlphaFold Protein Structure Database^29^. The atomic models were further refined against the density maps using Phenix.real_space_refine^50^ with the application of secondary structure restraints, geometry restraints and DNA-specific restraints. The quality of the final atomic models was evaluated by MolProbity^51^. Chimera, ChimeraX^52^ and PyMOL (http://pymol.org) were used for figure preparation.

### HMT assays

For a 20-μL end-point HMT reaction, 500 nM wild-type or mutants, 40 μM SAM, and 2 μM NCP^H2A^ or NCP^H2A.Z^ were mixed in the buffer of 25 mM HEPES-NaOH pH 7.5, 50 mM NaCl, 50 mM KCl, 5 mM MgCl_2_, 0.1 mg/mL BSA and 1 mM DTT, and were incubated at 32 °C for 15 min or 3 min. The reaction was stopped by adding 4 μL of 0.5% trifluoroacetic acid (TFA), and the HMT activity was evaluated using an MTase-Glo Methyltransferases Assay Kit (Promega). The luminescent signal that corresponds to the production of SAH was measured using a synergy H1 microplate reader in a white 96-well plate. Each reaction was run in triplicate, and is reported as the mean ± SD.

### MST assays

All MST-based experiments were performed on a Monolith NT115 Pico RED machine with 20% MST power and medium LED power. All of the proteins used in this assay have a 6× His-TEV tag at the N terminus. A Monolith His-tag Labeling Kit RED-tris-NTA (Nano Temper) was used to label the His-tagged proteins. For each reaction, 50 nM protein and 10 nM fluorescent dye were dissolved in 120 μL buffer containing 50 mM NaCl, 20 mM HEPES pH 7.5, 50 mM NaCl, 50 mM KCl, 1 mg/mL BSA, 0.05% Tween-20, 40 μM SAM and 2 mM DTT, and incubated at room temperature for 30 min. The nucleosomes were serially diluted in PCR tubes from 6 μM to 45.75 pM, then equal volumes of labeled proteins were added. After incubating for another 15 min, the samples were transferred into capillaries (Monolith NT115 Standard Treated Capillaries, MO-K002) in sequence. Dissociation constants (*K*_D_) were fitted with the MO Affinity Analysis software. Each reaction was run in triplicate.

### Mono-nucleosome IP

The nuclei were extracted with Nuclei Buffer (10 mM Tris-HCl, pH 7.5, 10 mM NaCl, 0.1% NP-40) containing Roche Complete Protease Inhibitor EDTA-Free. Then the chromatin was digested to mostly mono-nucleosomes by MNase (Sigma, N3755). Mono-nucleosomes were extracted by NB300 (10 mM Tris-HCl, pH 7.5, 300 mM NaCl, 0.1% Triton X-100) at 4 °C overnight. Flag-H2A or Flag-H2A.Z containing nucleosomes were immunoprecipitated by M2 Flag-agarose beads, and washed three times using NB300 buffer. Then the beads were boiled in 50 µL 1× SDS loading buffer and analyzed by western blot.

### Western blot aasay

Whole-cell extract samples were prepared by extracting cell pellet with Nuclei Lysis Buffer (10 mM Tris-HCl, pH 7.5, 10 mM NaCl, 0.1% SDS) with PMSF. The antibodies used were anti-H4K20me1 (Abcam, ab9051), anti-H4K20me2 (Abcam, ab9052), anti-H4K20me3 (Abcam, ab9053), anti-Myc (Cell Signaling Technology, 2276 S), anti-H4 (Cell Signaling Technology, 14149 S).

### ATAC-seq

ATAC-seq was performed according to Buenrostro et al.^53^ with minor modifications. Briefly, nuclei were extracted in Nuclei Buffer (10 mM Tris-HCl, pH 7.5, 10 mM NaCl, 3 mM MgCl_2_, 0.1% Triton X-100) with Roche Complete Protease Inhibitor EDTA-Free. After counting nuclei, 1× 10^5^ nuclei were incubated with 50 µL transposition mix containing 2 µL Tn5 transposase (Vazyme, TD711) at 37 °C for 30 min. Afterward, 50 µL 2× Stop buffer (20 mM EDTA, 0.2% SDS, 200 ug/mL Proteinase-K) was added to stop the reaction. The mixture was further incubated at 55 °C for 30 min, the DNA was extracted with Phenol:Chloroform:Isoamyl Alcohol (25:24:1), and precipitated by cold ethanol. 25 µL Tris-HCl buffer (10 mM Tris-HCl, pH 7.5) was used to resolve the DNA pellet, and 11.5 µL was used for Nextera library amplification. The library was purified with 1× AMPure XP beads (Beckman Coulter, A63881), and sequenced on DNBSEQ-T7 platform with PE150 mode.

### Sequencing data analysis

Paired-end reads were trimmed for adaptor sequence using cutadapt v4.3^54^ with parameters: -a CTGTCTCTTATACACATCT -A CTGTCTCTTATACACATCT -e 0.1 -n 2 -m 35 -q 30 –pairfilter = any, and then mapped to hg38 using Bowtie2 v2.5.1^55^ with parameters: -I 10 -X 1000 -3 5 --local --no-mixed --no-discordant --no-unal. Duplicates were marked using picard MarkDuplicates v2.27.5 (https://broadinstitute.github.io/picard/) with default parameters and removed using samtools view v1.17^56^ with parameters: -f 2 -F 1024 -q 10. ATAC peaks were detected with macs2 callpeak v2.2.7.1^57^ with parameter -f BAMPE -m 10 50. Signals within peaks were annotated using multiBigwigSummary, and heatmap was generated using plotHeatmap from deeptools v3.5.2^58^.

### Supplementary information

Supplementary information

## Data Availability

Cryo-EM maps of SUV420H1–NCP^H2A^ and SUV420H1–NCP^H2A.Z^ have been deposited in the Electron Microscopy Data Bank (EMDB) with accession codes EMD-36265 and EMD-36264, respectively. Atomic coordinates have been deposited in the Protein Data Bank (PDB) with accession codes 8JHG and 8JHF. High throughput sequencing data generated in this study were deposited to Gene Expression Omnibus (GEO) under accession GSE236249. All other data are available from the corresponding author upon reasonable request.
