# Supplementary material for: Structural basis of nucleosomal H4K20 recognition and methylation by SUV420H1 methyltransferase
Source: Cell Discov. 2023 Dec 5;9:120. doi: 10.1038/s41421-023-00620-5 (PMC10697951; doi:10.1038/s41421-023-00620-5)
Supplement: Supplementary file 1 — Supplementary information [file 41421_2023_620_MOESM1_ESM.pdf]

Supplementary Information

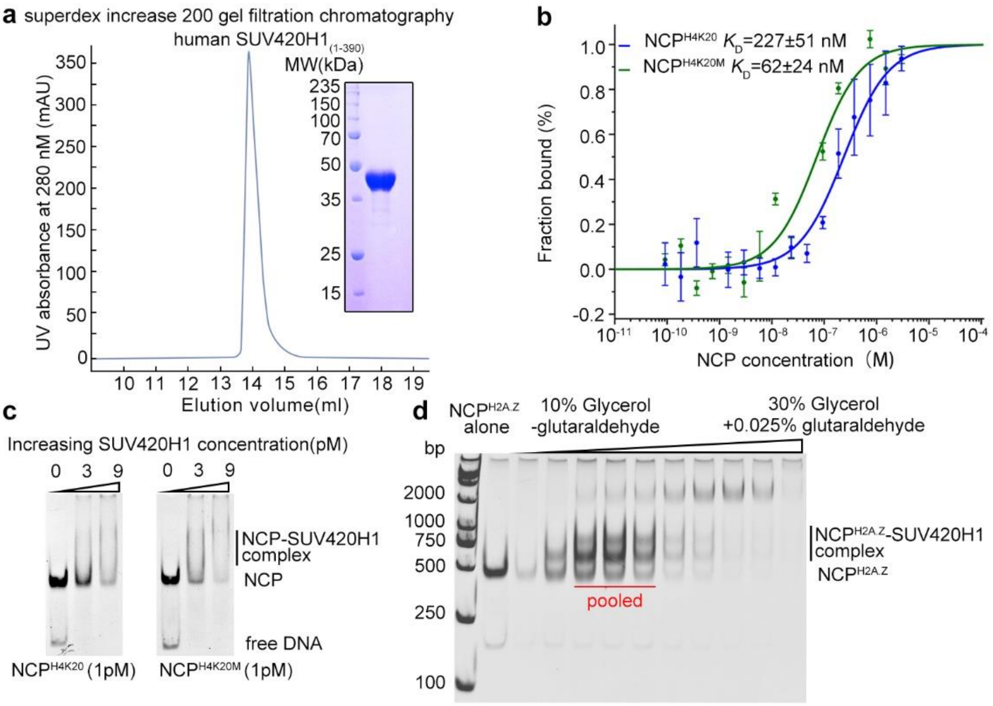

Supplementary Fig. S1 Purification of SUV420H1$_{(1\text{-}390)}$, EMSA analysis and GraFix fractionation of SUV420H1$_{(1\text{-}390)}$ in complex with nucleosomes. a, Gel filtration and SDS-PAGE analysis of the human SUV420H1$_{(1–390)}$. Experiments were repeated at least three times with similar results. **b,** MST binding assays of wild-type SUV420H1$_{(1–390)}$ on NCP^{H4K20} (K_D = 227 ± 51 nM) and NCP^{H4K20M} (K_D = 62 ± 24 nM). Binding curves and K_D values are also shown. Error bars represent mean ± SEM based on three independent measurements. **c,** EMSA analysis of SUV420H1$_{(1\text{-}390)}$ binding to NCP^{H4K20} and NCP^{H4K20M}. The concentrations of added SUV420H1 are indicated above the lanes. **d,** Representative images of 6% Native PAGE, stained with Gelred after GraFix fractionation. Fractions containing cross-linked species indicative of a protein complex were used in structural studies.

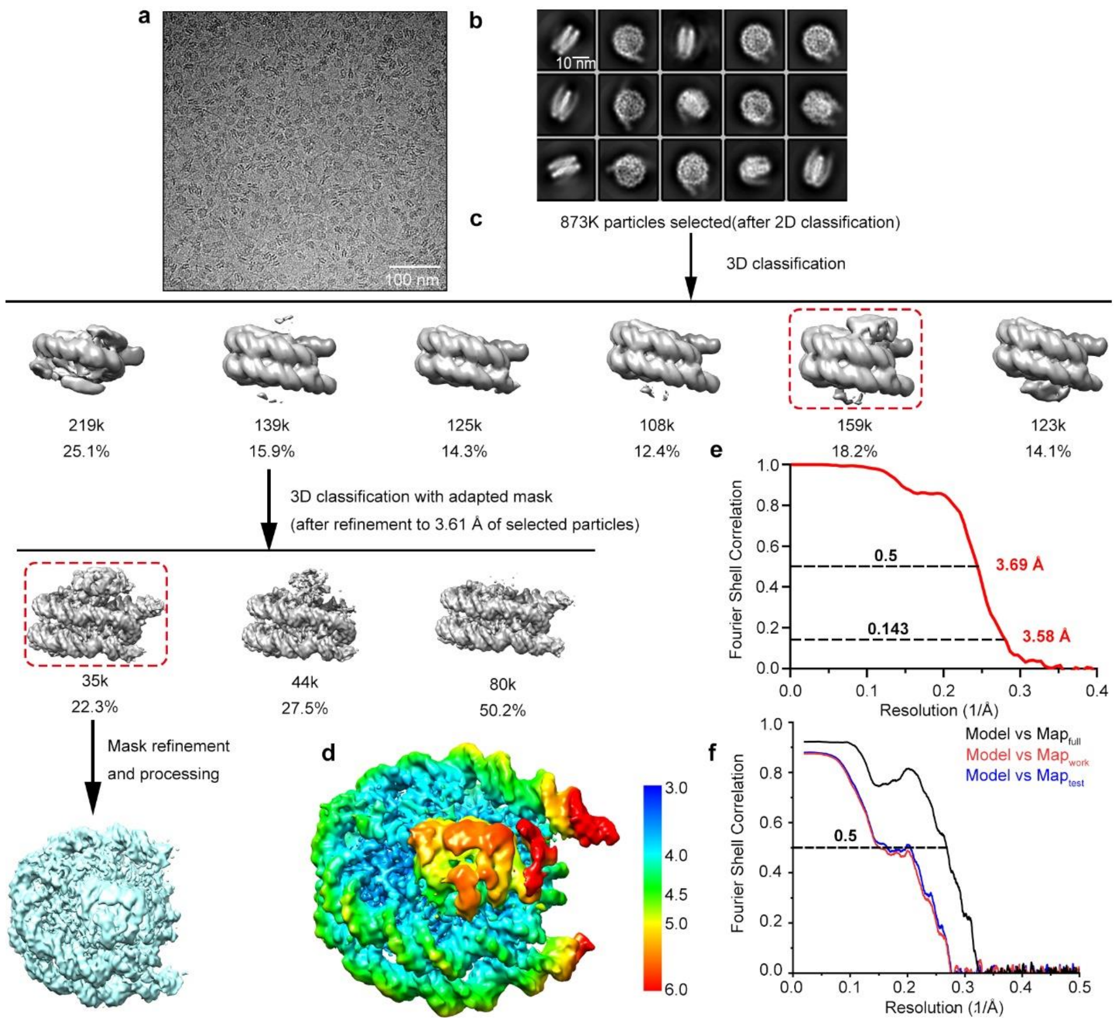

Supplementary Fig. S2 Cryo-EM structural analysis of SUV420H1$_{(1\text{-}390)}$ in complex with NCP^{H2A}. a, Representative cryo-EM micrograph from a total of 4,106 micrographs of the SUV420H1$_{(1\text{-}390)}$-NCP^{H2A} complex (low-pass-filtered to 20 Å). Scale bar, 100 nm. **b,** Selected 2D class averages of the SUV420H1$_{(1\text{-}390)}$-NCP^{H2A} complex. Scale bar, 10 nm. Box size 216, pixel size 0.92 Å. **c,** Workflow of the cryo-EM data processing procedures for the SUV420H1$_{(1\text{-}390)}$-NCP^{H2A} complex. It includes several rounds of 2D and 3D classification, refinement and masked refinement. **d,** Local-resolution map of the SUV420H1$_{(1\text{-}390)}$-NCP^{H2A} complex final density map. **e,** FSC curve of the SUV420H1$_{(1\text{-}390)}$-NCP^{H2A} complex final density map. The final resolution is 3.58 Å as determined by the FSC 0.143 criterion and 3.69 Å as determined by the FSC 0.5 criterion, respectively. **f,** Model-Map FSC curves calculated between the refined atomic model and the masked, sharpened half map used for refinement (Red, FSC_{work}), the

second masked, sharpened half-map not used in refinement (Blue, FSC_{test}) and the full, sharpened map (Black, FSC_{Full}).

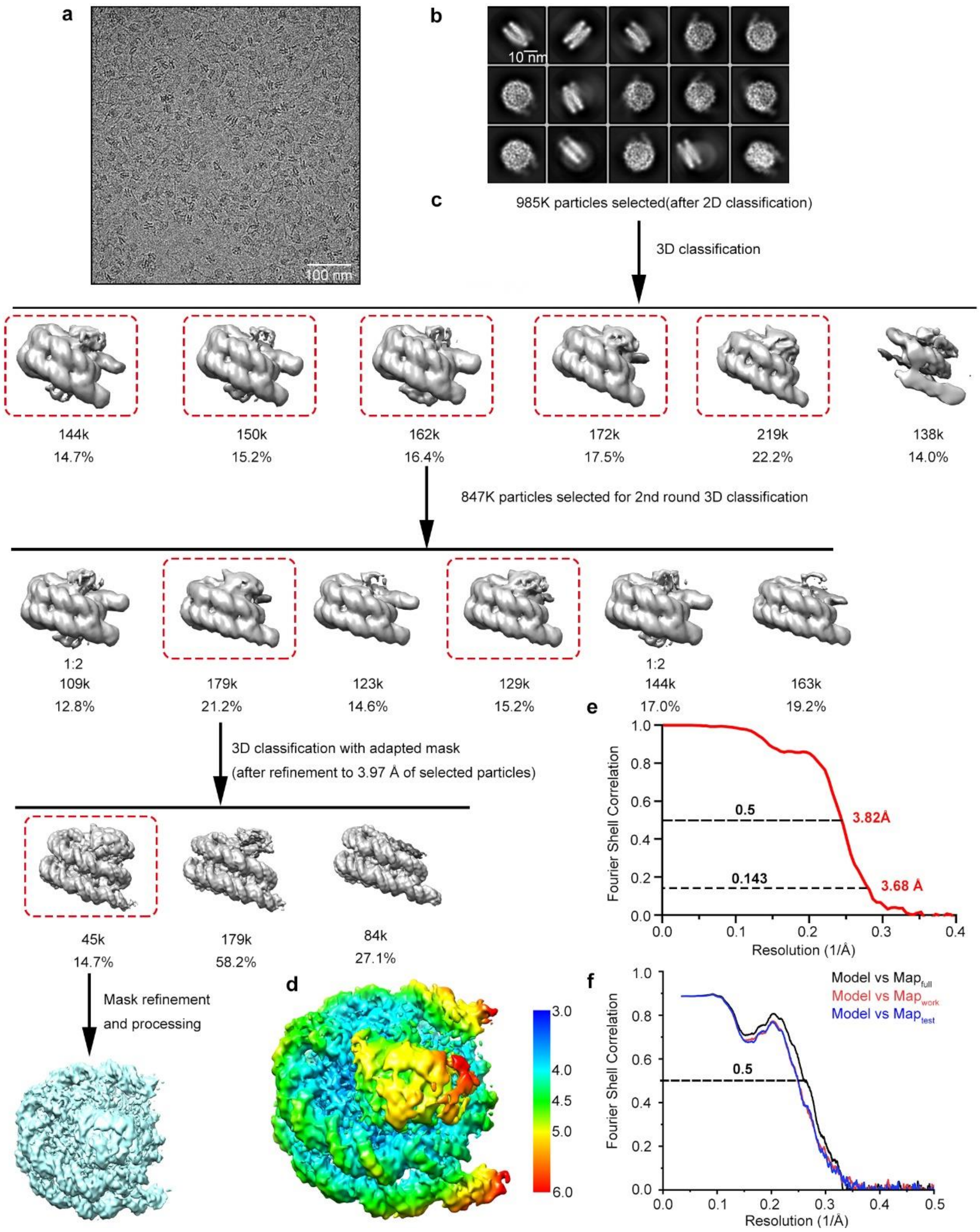

Supplementary Fig. S3 Cryo-EM structural analysis of SUV420H1$_{(1-390)}$ in complex with NCP$^{H2A.Z}$. a, Representative cryo-EM micrograph from a total of 6,915 micrographs of the SUV420H1$_{(1-390)}$-NCP$^{H2A.Z}$ complex (low-pass-filtered to 20 Å). Scale bar, 100 nm. **b,** Selected 2D class averages of the SUV420H1$_{(1-390)}$-NCP$^{H2A.Z}$ complex. Scale bar, 10 nm. Box size 216, pixel size 0.92 Å. **c,** Workflow of the cryo-EM data processing procedures for the SUV420H1$_{(1-390)}$-NCP$^{H2A.Z}$ complex. It includes several rounds of 2D and 3D classification, refinement and masked refinement. **d,**

Local-resolution map of the SUV420H1$_{(1-390)}$-NCP$^{H2A.Z}$ complex final density map. **e,** FSC curve of the SUV420H1$_{(1-390)}$-NCP$^{H2A.Z}$ complex final density map. The final resolution is 3.68 Å as determined by the FSC 0.143 criterion and 3.82 Å as determined by the FSC 0.5 criterion, respectively. **f,** Model-Map FSC curves calculated between the refined atomic model and the masked, sharpened half map used for refinement (Red, FSC$_{work}$), the second masked, sharpened half-map not used in refinement (Blue, FSC$_{test}$) and the full, sharpened map (Black, FSC$_{Full}$).

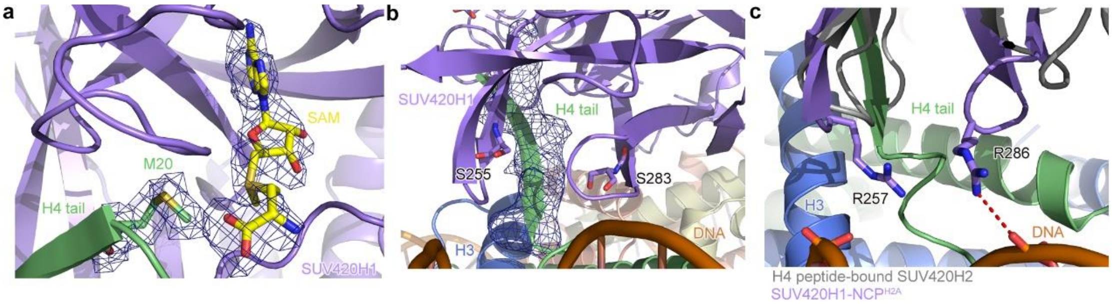

Supplementary Fig. S4 Cryo-EM density. a, The active site of SUV420H1^{SET} within the SUV420H1$_{(1\text{-}390)}$-NCP^{H2A} complex, shown with the EM density of the cofactor-product SAM and the substrate residue H4M20 in stereo mode. **b,** Detailed view of the location of SUV420H1 residues S255 and S283. The residues are shown as sticks and the EM density of the H4 tail residues 20-25 are shown as mesh. **c,** Alignment of the SUV420H2-H4 peptide structure (PDB code 4AU7, shown in grey) with SUV420H1$_{(1\text{-}390)}$-NCP^{H2A} complex structure.

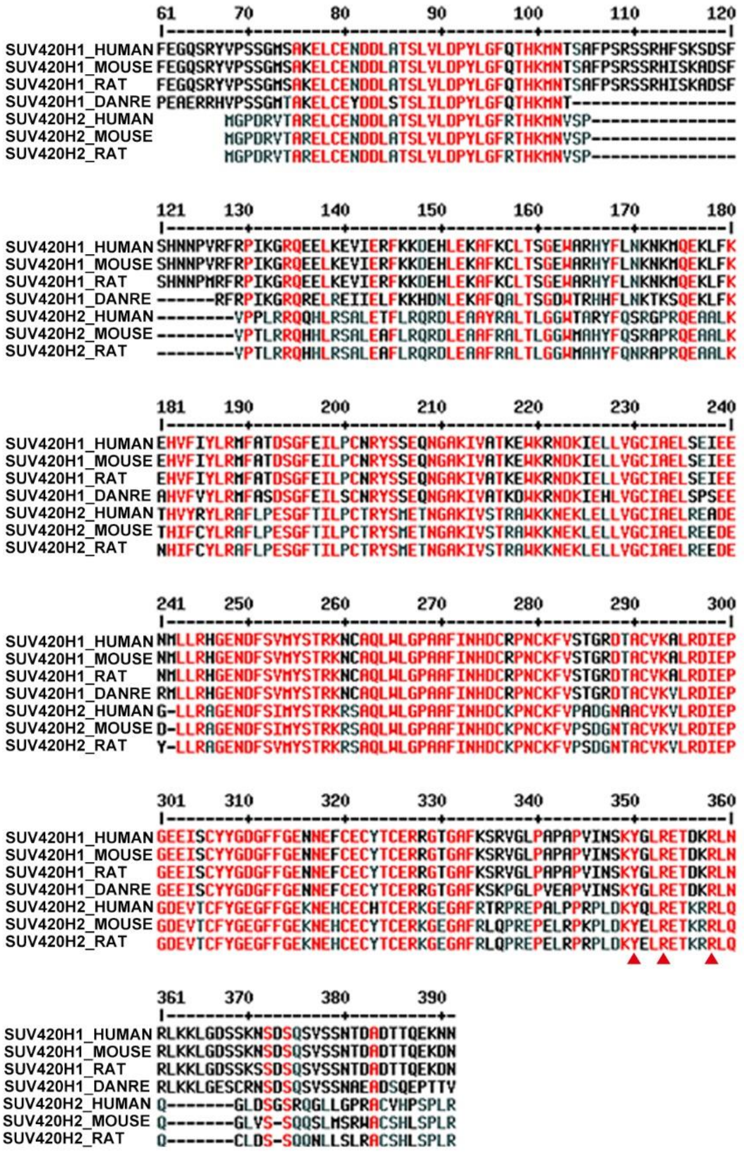

Supplementary Fig. S5 Multiple-sequence alignment of SUV420H1 and SUV420H2 across diverse species. The two arginine anchors R352, R357 and residue Y349 are highlighted.

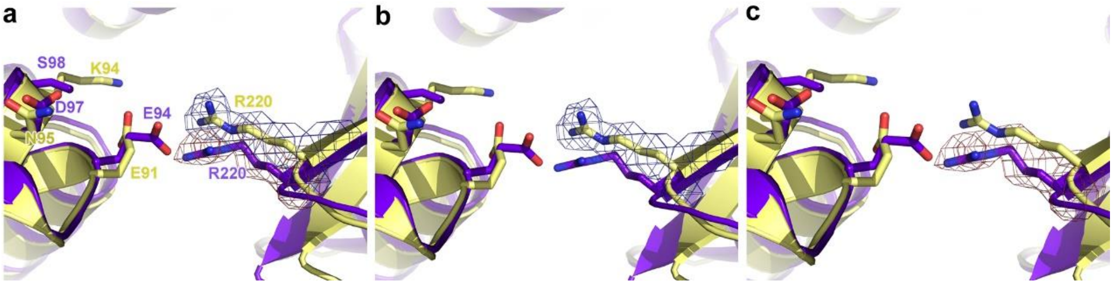

Supplementary Fig. S6 Detailed view of the electron microscopy density of R220 in SUV420H1$_{(1-390)}$-NCP^{H2A} and SUV420H1$_{(1-390)}$-NCP$^{H2A.Z}$ complexes. a, Alignment of SUV420H1$_{(1-390)}$-NCP^{H2A} and SUV420H1$_{(1-390)}$-NCP$^{H2A.Z}$ complexes showing the area comprised of R220 in SUV420H1 and acidic patch in nucleosome. SUV420H1$_{(1-390)}$-NCP^{H2A} is colored in yellow, SUV420H1$_{(1-390)}$-NCP$^{H2A.Z}$ is colored in purple. **b,** The EM density of R220 in SUV420H1$_{(1-390)}$-NCP^{H2A} complex is shown as mesh and contoured at 2σ level. **c,** The density of R220 in SUV420H1$_{(1-390)}$-NCP$^{H2A.Z}$ complex is shown as mesh and contoured at 2.5σ level.

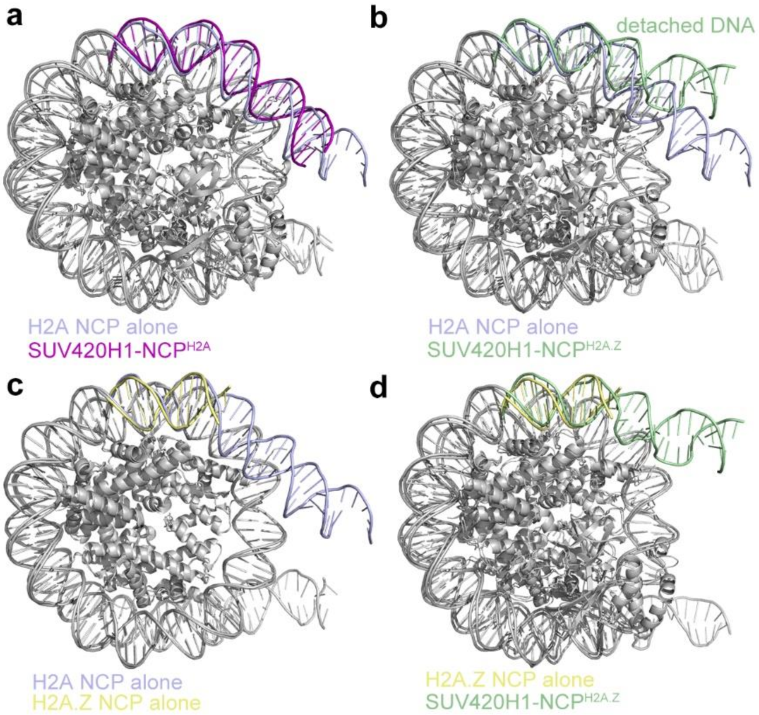

Supplementary Fig. S7 Overlapped nucleosome structures of H2A/H2A.Z alone nucleosomes and SUV420H1$_{(1-390)}$-bound H2A/H2A.Z nucleosomes. a, Overlapped nucleosome structures of H2A alone nucleosome (PDB code 7KTQ) and SUV420H1$_{(1-390)}$-bound H2A nucleosome. The DNA at SHL 6 and SHL 7 are colored in blue (H2A alone NCP) and purple (SUV420H1$_{(1-390)}$-bound H2A NCP), respectively. **b,** Overlapped nucleosome structures of H2A alone nucleosome and SUV420H1$_{(1-390)}$-bound H2A.Z nucleosome. The DNA at SHL 6 and SHL 7 are colored in blue (H2A alone NCP) and green (SUV420H1$_{(1-390)}$-bound H2A.Z NCP), respectively. **c,** Overlapped nucleosome structures of H2A alone nucleosome and H2A.Z alone NCP nucleosome (PDB code 7MIX). The DNA at SHL 6 and SHL 7 are colored in blue (H2A alone NCP) and yellow (H2A.Z alone NCP), respectively. **d,** Overlapped nucleosome structures of H2A.Z alone nucleosome and SUV420H1-bound H2A nucleosome. The DNA at SHL 6 and SHL 7 are colored in yellow (H2A.Z alone NCP) and green (SUV420H1$_{(1-390)}$-bound H2A.Z NCP), respectively.

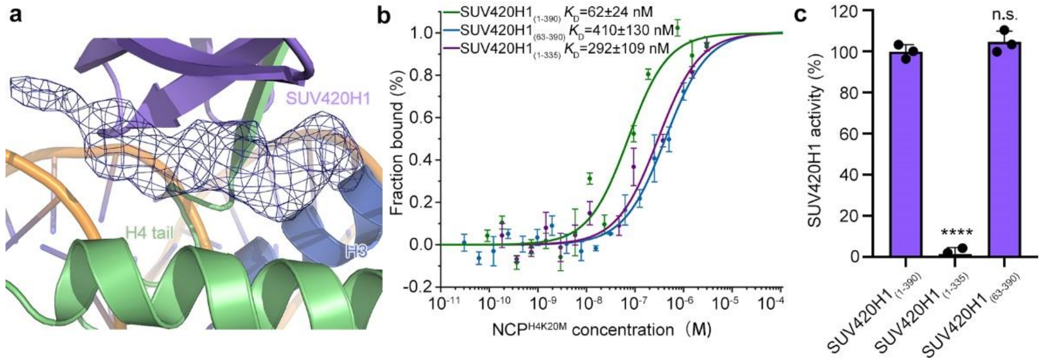

Supplementary Fig. S8 Cryo-EM density and biochemical assays of SUV420H1$_{(1\text{-}390)}$ and its truncations on nucleosomes *in vitro*. a, The unmodeled additional EM density in SUV420H1$_{(1\text{-}390)}$-bound H2A nucleosome as shown as mesh. **b,** MST assays of the binding to NCP^{H4K20M} of wild-type SUV420H1$_{(1\text{-}390)}$ (K_D = 62 ± 24 nM), SUV420H1$_{(1\text{-}335)}$ (K_D = 292 ± 109 nM) and SUV420H1$_{(63\text{-}390)}$ (K_D = 410 ± 130 nM). Binding curves and K_D values are also shown. **c,** Catalytic activity of wild-type SUV420H1$_{(1\text{-}390)}$ and SUV420H1$_{(1\text{-}390)}$ truncations on NCP^{H2A} by end-point HMT assays *in vitro*. Each assay was repeated at least three times with similar results. n = 3 independent experiments, two-tailed, unpaired t-test. **** $p < 0.0001$; n.s., not significant.

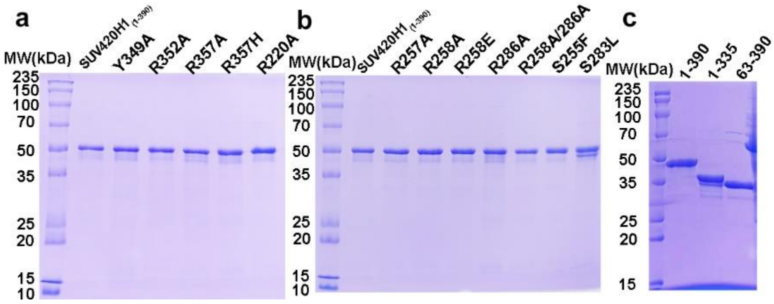

Supplementary Fig. S9 SDS-PAGE quantitative analysis of wild-type SUV420H1$_{(1-390)}$, SUV420H1$_{(1-390)}$ mutants and SUV420H1$_{(1-390)}$ truncations. a, b, SDS-PAGE gel shows the amounts of wild-type SUV420H1$_{(1-390)}$ and SUV420H1$_{(1-390)}$ mutants used in the end-point HMT assays. **c,** SDS-PAGE gel shows the amounts of wild-type SUV420H1$_{(1-390)}$ and SUV420H1$_{(1-390)}$ truncations used in the end-point HMT assays.

Supplementary Table S1

Cryo-EM data collection, refinement and validation statistics

	SUV420H1-NCP^{H2A} EMDB-36265 PDB 8JHG	SUV420H1-NCP$^{H2A.Z}$ EMDB-36264 PDB 8JHF
**Data collection and processing**		
Maginification	130,000	130,000
Voltage (kV)	300	300
Electron exposure (e-/Å^2)	50	50
Defocus range (μm)	1.0-2.0	1.0-2.0
Pixel size (Å)	0.92	0.92
Symmetry imposed	C1	C1
Initial particle image (no.)	873,129	984,971
Final particle image (no.)	35,448	45,377
Map resolution (Å) FSC threshold	3.58 (0.143)	3.68 (0.143)
**Refinement**		
Map sharpening *B* factor (Å^2)	-100	-100
FSC (half maps; 0.143) (Å)	3.69	3.66
FSC (model vs. full map; 0.5) (Å)	3.69	3.82
**Model composition**		
Protein residues	1040	1029
Ligands (SAM and Zn^{2+})	1 SAM, 1 Zn^{2+}	1 SAM, 1 Zn^{2+}
**R.m.s. deviations**		
Bond lengths (Å^2)	0.003	0.003
Bong angles (°)	0.570	0.557
**Validation**		
MolProbity score	1.81	1.73
Clash score	8.15	8.23
Poor rotamers (%)	0.00	0.00
**Ramachandran plot**		
Favored (%)	94.60	95.93
Allowed (%)	5.4	4.07
Disallowed (%)	0.00	0.00
